# Retinol Binding Protein, Sunlight Hours, and the Influenza Virus-Specific Immune Response

**DOI:** 10.3390/biomedicines10092322

**Published:** 2022-09-19

**Authors:** Nehali Patel, Rhiannon R. Penkert, Robert E. Sealy, Sherri L. Surman, Bart G. Jones, Karen Ringwald-Smith, A. Catharine Ross, Julia L. Hurwitz

**Affiliations:** 1 Department of Infectious Diseases, St. Jude Children’s Research Hospital, 262 Danny Thomas Place, Memphis, TN 38105, USA; 2 Department of Clinical Nutrition, St. Jude Children’s Research Hospital, 262 Danny Thomas Place, Memphis, TN 38105, USA; 3 Department of Nutritional Sciences, Pennsylvania State University, University Park, Pittsburgh, PA 16802, USA; 4 Department of Microbiology, Immunology and Biochemistry, University of Tennessee Health Science Center, Memphis, TN 38163, USA

**Keywords:** vitamins A and D, retinol binding protein, sunlight, month, influenza, antibody, immunoglobulin isotype

## Abstract

Healthy pediatric immune responses depend on adequate vitamin A and D levels. Relationships between solar ultraviolet B (UVB) radiation and vitamin D are well understood, while relationships between sunlight, vitamin A, and its serum escort, retinol binding protein (RBP), are not. A pediatric clinical study enrolled 2–8-year-old children at various times between September 2016 and March 2017, inclusive, in Memphis, Tennessee. A serum sample from each child was then assayed to examine the influence of season on vitamin levels. We found that RBP and RBP/retinol molar ratios decreased in winter months and RBP/retinol ratios correlated positively with the average daily sunlight hours per month. A food frequency questionnaire given to parents/guardians indicated a shift in dietary intake from plant-based foods to animal-based foods by children between winter and spring months. This translated to higher retinol and zinc (integral to RBP–transthyretin–retinol complexes) in the spring, perhaps explaining the seasonal influence on RBP/retinol. RBP and retinol were associated positively with IgG/IgM and IgA/IgM ratios. RBP and retinol, but not 25(OH)D, also correlated positively with influenza virus-specific antibodies. Retinol correlated negatively, while 25(OH)D correlated positively, with certain serum cytokine/chemokine levels. Significant differences in 25(OH)D, immunoglobulin ratios, and cytokines/chemokines were observed between black and white children. In sum, seasonal changes in dietary foods rich in retinol and zinc may have influenced RBP levels, which in turn influenced innate and adaptive immune responses. Results encourage routine monitoring and reporting of season, RBP, and vitamin levels in future clinical studies, as seasons may affect sunlight exposures, diet, vitamin levels, and immune protection against infectious disease.

## 1. Introduction

Healthy immune responses depend on vitamins A and D [1,2,3,4,5,6]. In geographical areas of the world where vitamin A intake is low, children often suffer from infectious diseases and can benefit from oral vitamin A supplementation [6]. In animal models of vitamin A deficiency, immune responses toward certain viral and bacterial vaccines or infections are weak, and can be improved with supplementation at the time of vaccination or infection [7,8]. Experiments with vitamin D have demonstrated both positive and negative influences on immune activities. In some cases, double deficiencies in vitamins A and D associate with the worst outcomes [9]. In other cases, a competition between the two vitamins may be at play [1,10,11].

Vitamins A and D are multi-functional and affect a variety of cell types [12,13]. As nuclear receptor ligands, vitamins A and D modulate nuclear receptors including the retinoic acid receptor (RAR) for vitamin A and the vitamin D receptor (VDR) for 25(OH)D. Nuclear receptors, in turn, influence gene configurations and gene expression [11]. Response elements (DNA motifs) to which vitamin A and D receptors preferentially bind appear throughout immunoglobulin and T cell receptor (TCR) loci. In fact, response element hotspots exist in switch sites necessary for class switch recombination (CSR) in the immunoglobulin heavy chain locus [14,15,16]. Direct binding of nuclear receptors to these sites, potentially modified by vitamin binding, will likely instruct gene rearrangement and transcription of immunoglobulin and T cell receptor genes [16]. The RAR and VDR form heterodimers with the same partner, RXR, and their response elements are similar, suggesting that receptors may frequently compete for DNA binding.

Vitamin A drives IgA production among activated B cells in vitro, and a previous clinical study in Memphis involving children and adults showed that RBP correlated with IgG4 and IgA [2,17,18,19,20]. These isotypes are expressed by constant region (C) genes positioned farthest downstream in the immunoglobulin heavy chain locus, illustrating support by retinoic acid for CSR among activated B cells. In contrast, in the Memphis study, 25(OH)D was positively correlated with serum IgM and IgG3 [2], isotypes encoded by upstream C genes that, in the case of IgM, are expressed in the absence of CSR.

Vitamin A levels are influenced primarily by diet, whereas vitamin D levels are influenced both by diet and solar ultraviolet B (UVB) radiation. The contribution of UVB radiation exposures to total serum vitamin D levels depends upon a plethora of factors including host diet, host age, host sex, host skin color, outdoor temperatures, season, latitude, altitude, cloud cover, surface reflection, and ozone [21]. Several different strategies have been used to measure solar radiation contributions to vitamin D status [21,22,23,24,25]. The effect of season on vitamin D synthesis is well appreciated [25], but relationships between season and levels of vitamin A, or its serum escort retinol binding protein (RBP), have been studied only minimally [26].

Here, we describe observational data from a small pediatric study (*n* = 44) that was originally designed to vaccinate children with an influenza vaccine and monitor changes in hemagglutination inhibition (HAI) titers [1,27]. Previous results from this study showed that (i) baseline levels of RBP correlated positively with improvements in HAI titers post-vaccination, (ii) baseline levels of 25(OH)D correlated negatively with improvements in HAI titers post-vaccination, (iii) supplemental vitamins were beneficial to improvements in HAI titers post-vaccination, but only when vitamin A and D levels were both low at baseline in study participants, and (iv) the month of study initiation influenced outcomes [1,27]. To further investigate the latter finding, we have now asked if levels of retinol, RBP, and 25(OH)D were influenced by the month of study enrollment. We also assessed relationships between vitamin A and D levels with baseline immunoglobulins and cytokines/chemokines.

An unexpected finding was that baseline RBP/retinol ratios were low during winter months and correlated with sunlight hours, perhaps due to seasonal changes in diet. Vitamin A levels correlated positively with baseline serum immunoglobulin patterns indicative of CSR, and with influenza virus-specific antibodies. Vitamin A levels correlated negatively with certain cytokines/chemokines, while 25(OH)D levels correlated positively. Results encourage scientists to monitor and report levels of RBP, retinol, 25(OH)D, and the month of sample collection as routine practices in future immunological research.

## 2. Materials and Methods

### 2.1. Study Design and Population

An observational study of 2–8-year-old male and female healthy children was conducted at St. Jude Children’s Research Hospital (St. Jude), as has been described previously (FLUVIT, ClinicalTrials.gov identifier NCT02649192, https://clinicaltrials.gov, first posted 7 January 2016, study start date 21 January, 2016) [1]. Informed consent was given by parents/guardians and assent was given by minors when age-appropriate.

Exclusion criteria included (i) chronic illness, (ii) history of a severe underlying condition, (iii) acute febrile or respiratory illness, (iv) developmental delay, (v) neurological disorder, (vi) current use of investigational or immunosuppressive drugs, antibiotics, or antivirals, and (vii) reported routine use of daily vitamin supplements. 

Our focus was on the 2016–2017 influenza season when 44 participants were enrolled (September 2016 to March 2017) [1,27]. Supplementary Table S1 lists the characteristics of these study participants. There were 28 females and 16 males. There were 31 black children and 13 white children enrolled. The protocol was reviewed and approved by the Institutional Review Board (IRB) of St. Jude. Baseline blood samples were examined in this study for serum retinol, RBP, 25(OH)D, immunoglobulin isotypes, influenza virus-specific antibodies, and cytokines/chemokines. The FLUVIT study involved influenza vaccinations, but the blood samples described in this report were taken at baseline only, prior to vaccinations. Children did not fast prior to blood collection. The blood samples collected for retinol studies were preserved in the cold and dark and were freeze-thawed once before testing. A second part of the study was optional and occurred on a later visit from that of the child’s blood draw. This involved a food frequency questionnaire (FFQ) answered by parents/guardians of enrolled children. Volunteers representing 30 enrolled children completed the FFQ.

### 2.2. Vitamin and RBP Analyses

Retinol was measured by extraction from test samples under conditions of UV-blocked lighting, using a previously described reverse-phase HPLC with photodiode array detection at 325 nm [1]. A retinol standard curve was used and the retinol molecular weight 286.44 was used for unit conversion. Briefly, aliquots of sera were saponified to convert vitamin A to unesterified retinol, then extracted into hexane. An aliquot of the internal standard, trimethylmethoxyphenyl-retinol, was added and the extract was evaporated to dryness under nitrogen. Samples were reconstituted in 100 µL methanol. Aliquots were injected onto a C-18 column and eluted with a mobile phase of 95% methanol–5% water. The limit of detection was below 0.2 µM plasma and the CV was <5%. As described previously [1], RBP was measured using a human RBP4 Quantikine kit (R&D Systems, Minneapolis, MN, USA). An RBP molecular weight of 21,400 was used for unit conversion. Vitamin D (25(OH)D) levels were tested using a Roche Elecsys electroluminescence assay that measured vitamins D2 and D3. A Roche Cobas 6000 e601 analyzer (CV ≤ 5.5, SD ≤ 1.1) was used.

The average retinol level was 1.21 µmol/L (range 0.61–1.95 µmol/L). The average RBP level was 1.04 µmol/L (range 0.45–1.52 µmol/L). The average RBP/retinol ratio was 0.87 (range 0.58–1.13). The average 25(OH)D level was 28.59 ng/mL (range 14.29–47.91 ng/mL). Black and white participants exhibited significant differences in 25(OH)D levels (see below) [21], but not in RBP or retinol levels. Details are provided in Supplementary Table S1. There were relatively high vitamin levels in some study participants; while these could have been due to oral vitamin supplementation, the reporting by parents/guardians of routine vitamin supplementation was an exclusion criterion for this study. The 25(OH)D levels were not well correlated with retinol for the total population (Supplementary Figure S1A) or for black and white children tested separately (Supplementary Figure S1B,C).

### 2.3. Immunoglobulin Analyses

Immunoglobulin isotypes were quantified among 40 of the 44 baseline samples with a bead-based multiplex immunoassay (Millipore Sigma, Billerica, MA, USA) using a Luminex 200 Multiplexing Instrument with xPONENT software (Luminex, Austin, TX, USA) and analyzed using Milliplex Analyst software (Millipore Sigma, Billerica, MA, USA). The tested isotypes included IgM, IgG1, IgG2, IgG3, and IgA. We analyzed each non-IgM value as a ratio with IgM to monitor immunoglobulin isotype switching by B cells. Influenza virus B-specific antibodies were measured with a human anti-influenza virus B IgG ELISA Kit (Ab108746 Abcam, Cambridge, MA, USA) per manufacturer’s instructions.

### 2.4. Cytokine Analyses

Forty of the 44 baseline serum samples were measured for 38 cytokines/chemokines using a Multiplex (Millipore MAP Kit cat #HCYTMAG-60K-PX38) and Luminex 200 Multiplexing Instrument. Milliplex Analyst software was used for data analyses. Cytokines/chemokines included EGF, eotaxin, FGF-2, FKN, Flt-3L, G-CSF, GM-CSF, GRO, IFNa2, IFNγ, IL-1α, IL-1β, IL-1RA, IL-2, IL-3, IL-4, IL-5, IL-6, IL-7, IL-8, IL-9, IL-10, IL12-p40, IL12-p70, IL-13, IL-15, IL-17a, IP-10, MCP-1, MCP-3, MDC, MIP-1α, MIP-1β, sCD40L, TGFα, TNFα, TNFβ, and VEGF. When factors gave scores at the lower or upper limit of detection, the limit of detection value was assigned for statistical analyses.

### 2.5. FFQ Dietary Assessments

On a separate visit from that of the child’s blood draw, parents/guardians were offered an FFQ to assess pediatric macronutrient and food group intake in Memphis. Trained members of the Clinical Nutrition Department of St. Jude administered the FFQ (NutritionQuest’s 2004 Block Questionnaire designed for ages 2–7). The questionnaire was developed with reference to the National Health and Nutritional Examination Survey (NHANES). Data analyses by NutritionQuest were with reference to USDA nutrient data. Data were viewed with attention to the seasons in which the FFQs were administered (winter: December, January, February of 2016–2017; spring: March, April, May of 2017). Thirty of the 44 enrolled children were represented in this voluntary FFQ study.

### 2.6. Sunlight Hour Measurements

Average sunlight hours per day for each study month were determined using data from the National Oceanic and Atmospheric Administration (NOAA, website: https://www.ncdc.noaa.gov/cdo-web/datasets/LCD/stations/WBAN:13897/detail, accessed 10 June 2022 [28]). Reports included daily times of sunrise and sunset in Tennessee (Nashville) between September 2016 and March 2017.

### 2.7. Statistical Analyses

Statistical tests were performed with Excel software (for Student’s *t*-tests evaluating FFQ results) or Graphpad Prism (for Mann–Whitney and correlation tests of FFQ and levels of vitamins, immunoglobulins, and cytokines/chemokines). Simple linear regression lines and quadratic regression curves were drawn with GraphPad prism software. Because the study was preliminary and observational, *p*-values were not adjusted for multiple comparisons.

## 3. Results

### 3.1. Serum RBP/Retinol Ratios Correlate with Sunlight Hours

**Figure 1:**
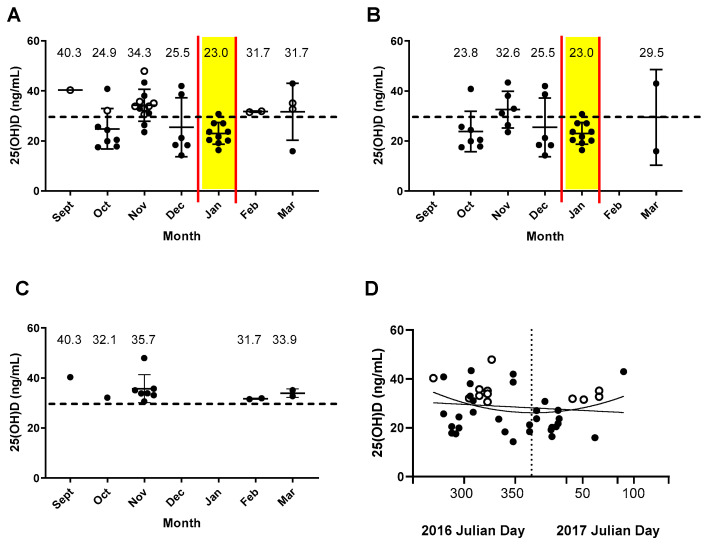
The 25(OH)D levels are shown as categorized by month for (**A**) all study participants (solid and open symbols represent black and white participants, respectively), (**B**) black participants alone, and (**C**) white participants alone. The month with the lowest mean value is highlighted in yellow. Means (also recorded by number above symbols) with standard deviations are shown. (**D**) 25(OH)D levels were plotted against Julian day of the year with solid and open symbols representing black and white participants, respectively. A linear regression line and a quadratic regression curve are shown (R^2^ = 0.01, *p* = 0.44 for the linear regression; R^2^ = 0.07 for the quadratic regression).

A study was performed with sera from 2–8-year-old healthy children to determine if serum vitamin levels were influenced by season. In Figure 1 are shown 25(OH)D levels, as categorized by month of serum collection. These are shown for the total population (Figure 1A), black children alone (B), or white children alone (C). The 25(OH)D levels were also plotted against Julian days of the year (D). Black participants scored significantly lower than white participants for 25(OH)D levels (Mann–Whitney test, *p* = 0.0009) and all levels less than 30 ng/mL were among the black children. In this small population, 25(OH)D levels trended lowest in January for the total population and for the black population (A–B), but levels below 30 ng/mL were observed in every month when there were at least three participants per month.

**Figure 2:**
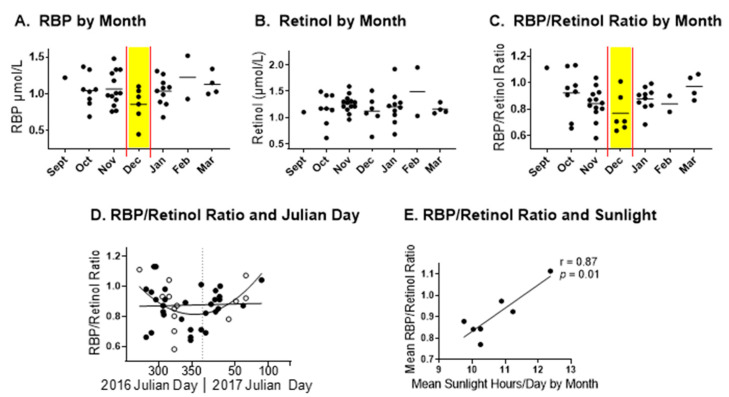
Seasonal changes in retinol/RBP ratios. (**A**) Individual RBP values (μmol/L) and means are shown per month of study enrollment. The month with the lowest mean value is highlighted in yellow. (**B**) Individual retinol values (μmol/L) and means are shown per month of study enrollment. (**C**) RBP/retinol molar ratios and means are shown per month. The month with the lowest mean ratio is highlighted in yellow. (**D**) RBP:retinol molar ratios were correlated with Julian days of the year. A linear regression line and a quadratic regression curve are shown (R^2^ = 0.001, *p* = 0.81 for the linear regression; R^2^ = 0.20 for the quadratic regression). Solid and open symbols represent black and white participants, respectively. Each symbol represents a different study participant for (**A**–**D**). (**E**) Mean RBP:retinol molar ratios were correlated with mean sunlight hours per day as measured in Nashville, Tennessee, each month (Pearson correlation).

A surprising result was observed when retinol and RBP were analyzed (Figure 2). RBP levels were relatively low in the winter, with the lowest mean values in December (A). Retinol levels were more stable (B), and RBP/retinol ratios were consequently low in December (C–D). In this case, no difference between black and white participants was observed. RBP/retinol mean ratios were significantly correlated with the average sunlight hours per day in Tennessee by study month (September to March, E).

### 3.2. FFQs Indicate Changes in Diet between Winter and Spring Months

To explain seasonal changes in RBP and RBP/retinol, we analyzed a NutritionQuest FFQ that was given to parents/guardians during a separate visit after pediatric samples were collected. The FFQ analysis was used with an understanding of limitations associated with diet recall. The parents/guardians of 30 children volunteered to complete the FFQ between December of 2016 and May of 2017.

**Figure 3:**
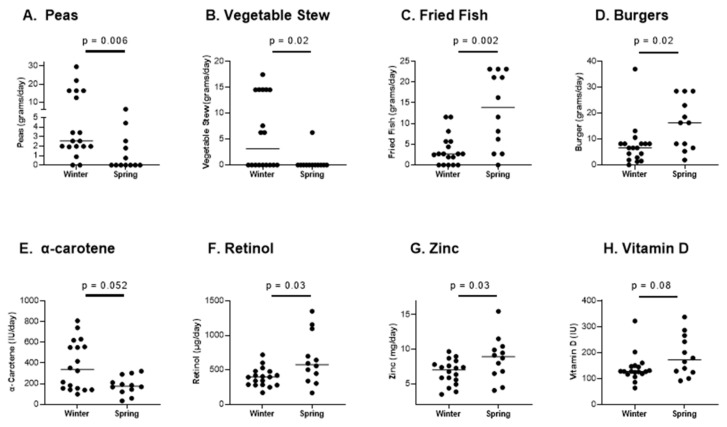
Dietary intake changes with season. Parents/guardians of 30 children completed an FFQ. FFQ results reported in the winter (December (2016), January (2017), and February (2017)) were compared to results reported in the spring (March to May 2017). A portion of variables are shown, including (**A**–**D**) food items, and (**E**–**H**) calculated intakes of vitamins and zinc. Each symbol represents a different child with medians shown. There were seven foods (from approximately 90 tested food groups) that were significantly different between winter and spring, as revealed both by *t*-tests and Mann–Whitney tests. These were peas, vegetable stews, peanut butter, butter, and pretzels (more prevalent in the winter), and fried fish and burgers (more prevalent in the spring). Results of Mann–Whitney tests are shown. Significant differences between black and white participants were not observed for these variables.

Results of the FFQ (Figure 3) were grouped by the season of completion, either winter (December, January, February 2016/2017) or spring (March, April, May 2017). Among approximately 90 food groups queried in the FFQ, there were seven responses that were significantly different between winter and spring months, as identified both by *t*-tests (Excel software) and Mann–Whitney tests (GraphPad Prism software).

As shown in Figure 3A,B, the plant-based foods peas (*p* = 0.006) and vegetable stews (*p* = 0.02) were more frequently consumed in the winter compared to the spring. Three other foods with greater intakes in the winter were peanut butter (*p* = 0.03), butter (*p* = 0.047), and pretzels (*p* = 0.01). In contrast, fried fish (*p* = 0.002) and burgers (*p* = 0.02) were more frequently consumed in the spring (Figure 3C,D).

Additional food entries that were significantly different between winter and spring months based on *t*-tests alone (Excel software) were carrots and bean soup (g/day, higher in winter), cups of deep yellow-orange vegetables and starchy vegetables (higher in winter), and intake frequencies of processed cheese and Lunchables (higher in spring). Overall, the diets of this pediatric population shifted from a bias toward plant-based foods in the winter to animal-based foods in the spring.

We next examined the FFQ-calculated intakes of pertinent vitamin/minerals including vitamin A, vitamin D, and zinc (the latter of which supports RBP function). The calculated alpha carotene intake trended higher in the winter (Figure 3E, for alpha carotene, *p* = 0.052; for beta carotene, *p* = 0.36), reflecting the higher consumption of vegetables. In contrast, calculated retinol intakes (by μg/day) were significantly higher in the spring (Figure 3F). The inverse trends of α-carotene and retinol had a balancing effect in that there was no significant difference in total calculated vitamin A intakes (IU) between winter and spring. Zinc intakes were significantly higher in the spring compared to the winter, reflective of the increased burger intake (Figure 3G). Calculated vitamin D intake values trended higher in the spring, but were not significantly different between seasons (Figure 3H).

### 3.3. Vitamins, RBP, and Antibody Levels in Pediatric Sera

**Figure 4:**
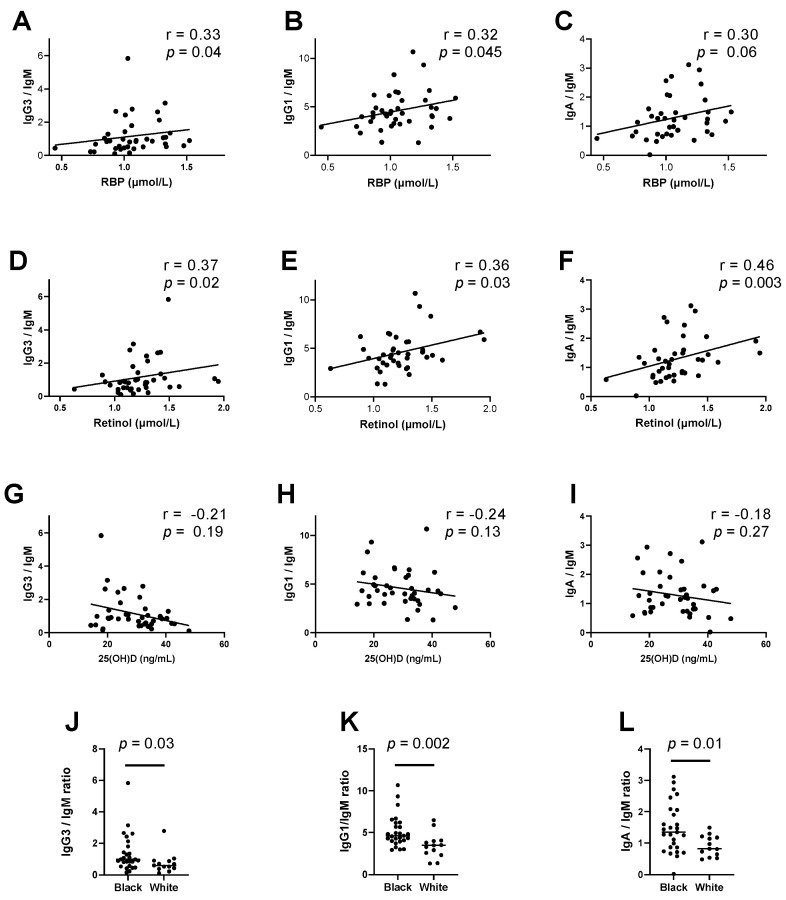
Vitamin levels correlate with serum immunoglobulin isotype patterns. (**A**–**I**) RBP and retinol, but not 25(OH)D, correlate positively with isotype ratios, indicative of immunoglobulin class switch from IgM to non-IgM isotypes. Each symbol represents a different participant. A simple linear regression line is plotted. Spearman correlation test results are shown. (**J**–**L**) Black and white children differed significantly in isotype ratios. Medians and results from Mann–Whitney tests are shown.

Based on previous demonstrations that vitamin A and D levels were associated with distinct immunoglobulin isotype expression patterns [2], we examined antibody isotypes as a function of RBP, retinol, and 25(OH)D levels (Figure 4A–I).

Retinol correlated negatively with IgM (Spearman’s rank correlation coefficient, r = −0.34, *p* = 0.03), while 25(OH)D correlated negatively with IgG1 (r = −0.32, *p* = 0.04), suggesting that retinol, more so than 25(OH)D, supported CSR and the switch from IgM to non-IgM isotypes. RBP and retinol, but not 25(OH)D, correlated positively with isotype ratios including IgG3/IgM, IgG1/IgM, and/or IgA/IgM (Figure 4A–F). Values for IgG2/IgM ratios also trended higher as retinol and RBP increased, but these correlations were not statistically significant (Spearman’s correlation for retinol, r = 0.30, *p* = 0.06; Spearman’s correlation for RBP, r = 0.24, *p* = 0.13). In contrast, there was a trend toward negative correlations between 25(OH)D levels and the same isotype ratios (Figure 4G–I). Consistent with these negative trends and with the lower levels of 25(OH)D in black children compared to white children, there were significantly higher isotype ratios for IgG3/IgM, IgG1/IgM, and IgA/IgM in black children compared to white children (Figure 4J–L).

**Figure 5:**
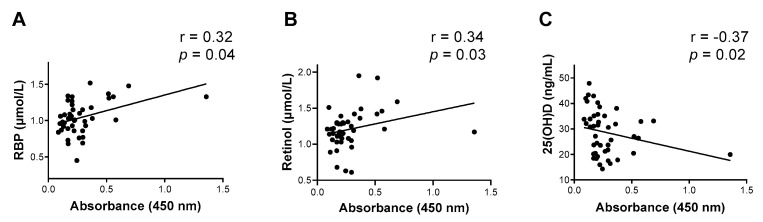
RBP and retinol, but not 25(OH)D, correlate with influenza virus B-specific binding antibodies in pediatric human sera. (**A**) RBP, (**B**) retinol, and (**C**) 25(OH)D are each plotted as a function of baseline scores for influenza virus B-specific binding antibodies. Each symbol represents a different participant. A simple linear regression line is plotted. Spearman correlation test results are shown.

RBP and retinol were associated positively with influenza virus B-specific IgG antibodies, while 25(OH)D was negatively correlated with these antibodies (Figure 5A–C). There was not a significant difference between black and white children when influenza virus B-specific antibodies were evaluated.

### 3.4. Vitamins, RBP, and Cytokine/Chemokine Levels in Pediatric Sera

**Figure 6:**
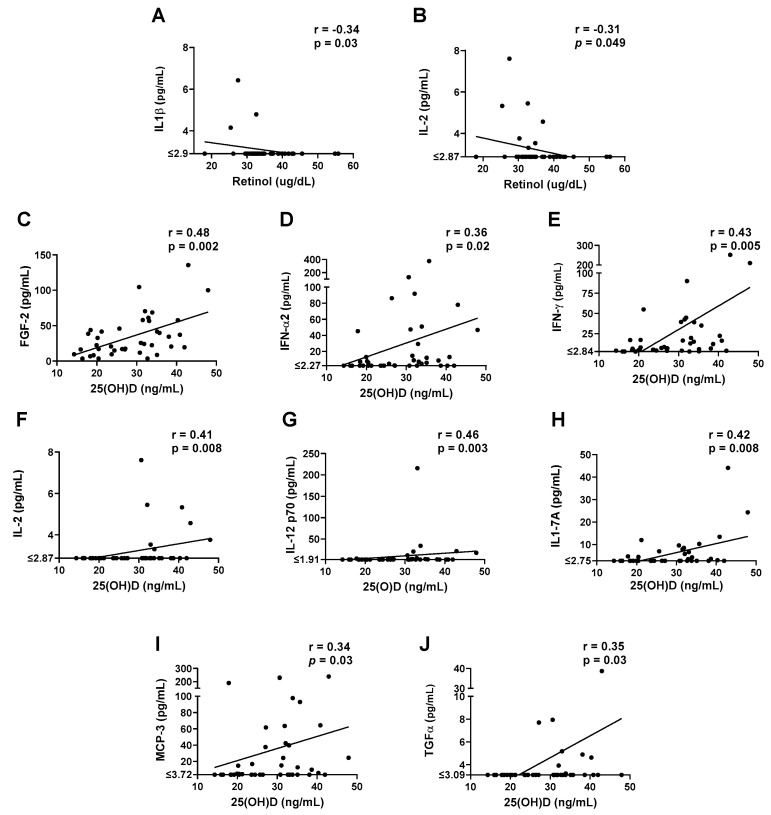
Vitamin and cytokine/chemokine levels. Significant correlations are shown between cytokine/chemokine (**A**,**B**) and retinol (**C**–**J**) or 25(OH)D levels. Each symbol represents a different individual. Spearman correlation test results are shown.

We next examined cytokines/chemokines as a function of RBP, retinol, and 25(OH)D levels. As shown in Figure 6, there were two significant negative correlations between cytokine/chemokine and retinol levels (Figure 6A,B). Again, there was an opposite influence of 25(OH)D in that there were eight positive correlations between cytokine/chemokine and 25(OH)D levels (Figure 6C–J).

**Figure 7:**
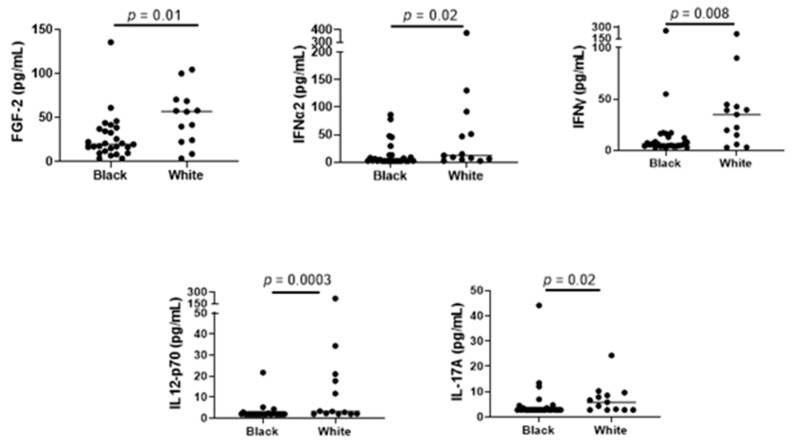
Cytokine/chemokine levels in black and white children. Black and white children were compared for the nine cytokines/chemokines shown in Figure 6. In five cases, white children exhibited higher values than black children, consistent with their higher serum 25(OH)D levels. Each symbol represents a different individual. Median and Mann–Whitney results are shown. Levels of IL-1β, IL-2, MCP-3, and TGFα did not differ significantly between the black and white populations.

A comparison of black and white children showed that for several cytokines/chemokines shown in Figure 6, black children had significantly lower levels than white children (Figure 7). This was consistent with the positive correlation between cytokines and 25(OH)D, and the lower 25(OH)D levels in black children compared to white children.

## 4. Discussion

### 4.1. An Unexpected Correlation between RBP/Retinol and Sunlight Hours

Our study was performed to examine influences of vitamin levels on seasonal changes in immune function [27]. An expected result was that 25(OH)D levels were low in winter months, consistent with lower UVB radiation exposures [13]. An unexpected result was that RBP was also lower in winter months in our study population. Data showed that the molar ratio of RBP and retinol, predicted to be a constant of 1:1 [29], was actually subject to change and correlated positively with average daily sunlight hours in our study location. To explain shifts in RBP, we used data from an FFQ, and observed changes in diets between winter and spring months. Plant-based foods, including peas, vegetable stews, and peanut butter, were more frequently consumed in winter months, whereas animal-based foods, including fried fish and burgers, were more frequently consumed in the spring. These diet changes toward animal-based foods translated to increased retinol and zinc intakes in the spring. Zinc is known to support transthyretin–RBP–retinol complexes [30,31] and could thereby improve RBP/retinol ratios. It is noteworthy that in a separate report, a vegan diet in young children was associated with low RBP levels, suggesting that plant-based foods are weaker than animal-based foods in their support of serum RBP [32].

Changes in diet in Memphis by season may have been influenced by local traditions. The city of Memphis is world-renowned for barbecued foods and barbecue festivals during spring months. As temperatures warm and outdoor activities increase, traditional barbecues may promote a shift toward the consumption of animal-based foods [33].

### 4.2. Correlations of Vitamins with Adaptive and Innate Immune Factors

Vitamins A and D are each pertinent to healthy immune function. Vitamins can signal cells at the plasma membrane, within the cytoplasm, and within the nucleus [11,12,16,34,35,36]. Immune cells that are influenced by vitamins include B cells, T cells, and cells of the innate immune system, including dendritic cells and other antigen-presenting cells [4]. An enzyme required for vitamin A metabolism (ALDH1A2) is prominently expressed among cells in the respiratory tract and intestine where critical mucosal immune effectors reside [4,17].

Consistent with a previous study in Memphis [2], our results revealed positive correlations between retinol and RBP with IgG3/IgM, IgG1/IgM, and IgA/IgM, indicative of class switches from IgM to non-IgM isotypes. In contrast, there was a trend toward negative correlations between 25(OH)D and the same isotype ratios. Opposite results for retinol and 25(OH)D were also observed when serum cytokines/chemokines were measured. Retinol correlated negatively with two factors (matching previous reports of inflammation inhibition by retinol [2,37]), whereas 25(OH)D correlated positively with eight factors. These seemingly contrasting correlations for the two vitamins may be an outcome of the competition described above. Retinol and 25(OH)D may exert unique influences on antibody and cytokine expression, and may cross-compete for function.

Our analyses of participants in the FLUVIT study previously showed that baseline RBP levels correlated positively with, and 25(OH)D correlated negatively with, immune responses toward influenza virus vaccines [1]. Here, we present data showing that RBP and retinol, but not 25(OH)D levels, correlated positively with influenza virus B-specific binding antibodies at study baseline. Perhaps 25(OH)D supported IgM expression whereas retinol prompted an immunoglobulin class switch from IgM to IgG or IgA, the latter providing additional benefit to the influenza virus-specific immune response.

### 4.3. Serum Immunoglobulin and Cytokine/Chemokine Differences between Black and White Pediatric Populations

The significantly lower 25(OH)D levels in black children compared to white children translated to differences in immunoglobulin isotype and cytokine/chemokine patterns. There were higher IgG/IgM and IgA/IgM ratios in black children (possibly due to improved retinol function when 25(OH)D is low [11]), and reductions in serum cytokine/chemokine levels. Each of these parameters may ultimately influence human protection against pathogens and consequent inflammatory responses.

### 4.4. Study Limitations

We note that there were several limitations to our observational study. The study was limited by a small sample size. A single location was studied and a single serum sample was analyzed for each study participant. Changes in diets with season may have been idiosyncratic and may not be applicable to other pediatric populations. For example, the shifts in diet from winter to spring were likely influenced by local traditions in Memphis. As another example, we found that there were 25(OH)D levels below 30 ng/mL in every month for which there were more than three participants, and 25(OH)D levels trended lowest in January [38]. A study of several thousand British adults identified the lowest 25(OH)D levels in February [39], and a study of results from more than two million adults in the United States revealed a trough in late February or early March [40]. In our study, we measured sunlight hours and did not measure UVB solar radiation or the many factors that influence radiation-mediated vitamin D synthesis [21,41]. Again, we recognize that numerous variables influence vitamin levels, including UVB exposures, host age, host sex, host weight, host diet, host behaviors, trafficking of factors among tissues, and factor half-lives [25,42,43,44].

Despite study limitations, data illustrated the potential effects of season on RBP/retinol ratios (RBP/retinol ratios are not precisely 1:1), an influence that has not been recognized previously and that deserves further evaluation. Seasonal changes, possibly in part due to changes in diet, may influence RBP, RBP/retinol ratios, 25(OH)D, and the adaptive/innate immune response. The month/season of study enrollment is clearly an important variable in clinical research [27].

### 4.5. Clinical Implications

Levels of vitamins A and D exert significant influences on pediatric and adult immune health. While cause–effect relationships are not always proven, low vitamin A levels have been associated with poor immune responses and/or severe disease consequences in numerous contexts, including (i) children with severe asthma [45], (ii) children with sickle cell disease [46,47], an animal model of cystic fibrosis [48], vitamin-A-deficient (VAD) mice following bacterial and/or viral infections [7,8,49,50], vaccinated children [1], and children with measles [5]. High levels of vitamin D have been associated with a decreased incidence of tuberculosis, in part due to the 25(OH)D-mediated enhancement of cathelicidin production by macrophages [13]. While clinicians are often observant of patient 25(OH)D levels and changes with season, less attention is given to retinol, RBP, and RBP/retinol ratios. Furthermore, reports of clinical study data rarely include the season/month of sample collection, meaning that an important variable pertinent to vaccines and infectious disease outcomes may be overlooked. Our demonstration that RBP/retinol ratios can fluctuate with season encourages clinicians/researchers to monitor and report month and vitamin levels, including serum RBP, as part of routine pediatric care and research. Possibly, when RBP/retinol ratios are discovered to be low, a change in diet could correct the deficit and thereby improve immune protection.

### 4.6. Conclusions

Previous research has indicated that seasons with high UVB radiation associated with improved vitamin D levels. Here, we show that RBP, an important escort for vitamin A, also fluctuated with season. The predicted RBP/retinol molar ratios (1:1) were not constant [29]. Instead, RBP/retinol ratios correlated with average daily sunlight hours per month, perhaps due to seasonal changes in diet among the tested children. We further demonstrated positive associations between RBP and retinol, but not 25(OH)D, with serum immunoglobulin isotype patterns indicative of an immunoglobulin isotype class switch, plus anti-influenza virus-specific antibodies. In addition, retinol correlated negatively, while 25(OH)D corelated positively, with certain cytokines/chemokines. Black and white participants differed significantly in 25(OH)D levels, immunoglobulin isotype ratios, and cytokine/chemokine levels. Taken together, results reveal a previously-unknown contributor to seasonal changes in immune health.

## Data Availability

Data are available upon request to the corresponding author.

## Supplementary Materials

The following supporting information can be downloaded at: https://www.mdpi.com/article/10.3390/biomedicines10092322/s1, Figure S1. Poor correlation between serum vitamin D and retinol levels. Each symbol represents a different study participant, comparing serum levels for 25(OH)D and retinol. Results are shown for (A) the total population (Spearman Rank Correlation r = 0.16, *p* = 0.29), (B) black children alone, and (C) white children alone; Table S1: Participant Characteristics.

## Abbreviations

RBP—retinol binding protein; ELISA—enzyme linked immunosorbent assay; FFQ—food frequency questionnaire; NHANES—National Health and Nutritional Examination Survey; CSR—class switch recombination.
