# Supplementary material for: Retinol Binding Protein, Sunlight Hours, and the Influenza Virus-Specific Immune Response
Source: Biomedicines. 2022 Sep 19;10(9):2322. doi: 10.3390/biomedicines10092322 (PMC9496108; doi:10.3390/biomedicines10092322)
Supplement: Supplementary file 1 [file biomedicines-10-02322-s001.zip › biomedicines-1823460-supplementary.pdf]

Table S1. Participant Characteristics									
ID	Enroll-ment Year	Enroll-ment Month	sex	age	race	Retinol (μmol/L)	RBP (μmol/L)	RBP/retinol	Vitamin D (ng/ml)
FV013	2016	Sept	Female	8	White	1.10	1.22	1.11	40.3
FV014	2016	Oct	Male	7	Black	1.42	0.93	0.66	25.7
FV015	2016	Oct	Male	2	Black	0.89	0.87	0.98	40.8
FV016	2016	Oct	Male	5	Black	1.42	1.37	0.96	20.5
FV017	2016	Oct	Male	7	Black	1.49	1.03	0.69	17.8
FV019	2016	Oct	Male	3	Black	0.61	0.69	1.13	17.5
FV020	2016	Oct	Female	5	Black	1.17	1.33	1.13	19.9
FV021	2016	Oct	Female	2	Black	1.17	1.06	0.91	24.4
FV022	2016	Oct	Male	2	White	1.14	1.06	0.93	32.1
FV023	2016	Nov	Male	4	Black	1.36	1.18	0.87	38.1
FV024	2016	Nov	Female	4	Black	1.21	1.01	0.83	32.9
FV025	2016	Nov	Female	8	Black	1.46	1.33	0.91	26.4
FV026	2016	Nov	Female	8	Black	1.30	1.28	0.98	31.1
FV027	2016	Nov	Male	3	Black	1.21	0.98	0.81	43.4
FV028	2016	Nov	Female	8	White	1.59	1.48	0.93	33.1
FV029	2016	Nov	Female	5	White	1.28	1.33	1.04	35.7
FV030	2016	Nov	Female	8	White	1.31	0.76	0.58	35.1
FV031	2016	Nov	Female	6	White	1.21	0.84	0.70	33.8
FV032	2016	Nov	Female	7	White	0.96	0.77	0.80	30.6
FV033	2016	Nov	Male	2	White	1.15	0.97	0.85	33.9
FV034	2016	Nov	Female	3	White	1.06	0.92	0.87	47.9
FV035	2016	Nov	Male	7	Black	1.29	1.01	0.78	23.6
FV036	2016	Dec	Female	4	Black	1.17	1.04	0.89	18.3
FV038	2016	Dec	Female	2	Black	0.63	0.45	0.71	14.3
FV039	2016	Dec	Male	3	Black	1.30	0.86	0.66	38.7
FV040	2016	Dec	Female	5	Black	1.51	0.96	0.64	42.0
FV041	2016	Dec	Female	2	Black	1.03	0.73	0.71	18.4
FV042	2016	Dec	Female	8	Black	1.08	1.10	1.01	21.2
FV045	2017	Jan	Female	3	Black	1.92	1.31	0.69	27.1
FV046	2017	Jan	Female	4	Black	1.05	0.86	0.82	23.7
FV047	2017	Jan	Female	3	Black	1.22	1.08	0.88	30.8
FV048	2017	Jan	Female	8	Black	1.39	1.27	0.91	19.2
FV049	2017	Jan	Female	2	Black	0.91	0.89	0.97	20.2
FV050	2017	Jan	Male	6	Black	1.19	0.99	0.83	16.4
FV051	2017	Jan	Male	4	Black	1.28	1.09	0.85	20.4
FV053	2017	Jan	Female	5	Black	1.25	1.15	0.91	21.7
FV054	2017	Jan	Female	6	Black	1.13	1.04	0.93	27.1
FV055	2017	Jan	Female	6	Black	0.68	0.68	1.00	23.7
FV057	2017	Feb	Male	7	White	1.95	1.52	0.78	31.9
FV058	2017	Feb	Female	8	White	1.03	0.93	0.90	31.5
FV060	2017	March	Female	6	Black	1.15	1.00	0.87	16.0
FV061	2017	March	Female	4	White	1.11	1.03	0.92	35.1
FV062	2017	March	Male	5	White	1.08	1.15	1.07	32.7
FV064	2017	March	Male	2	Black	1.29	1.34	1.04	43.0

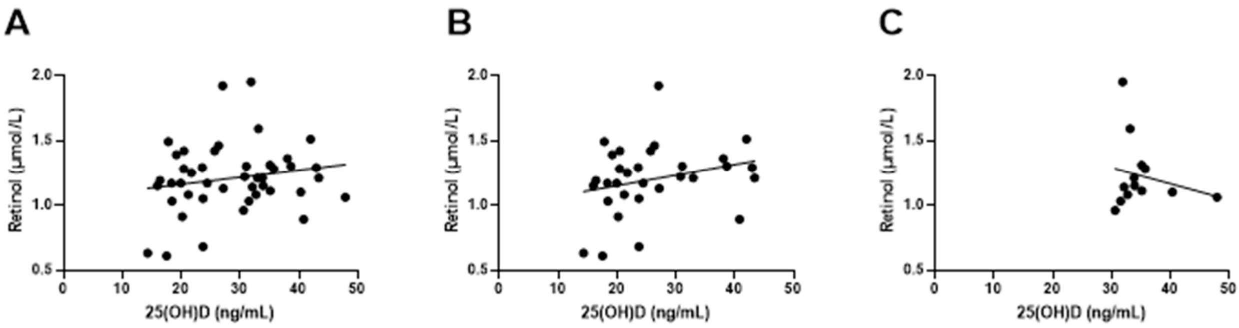

Supplementary Figure S1. Poor correlation between serum vitamin D and retinol levels. Each symbol represents a different study participant, comparing serum levels for 25(OH)D and retinol. Results are shown for (**A**) the total population (Spearman Rank Correlation r = 0.16, p = 0.29), (**B**) black children alone, and (**C**) white children alone.
